# Crystal structure of 2-[(3a*S*,6*R*)-3,3,6-trimethyl-3,3a,4,5,6,7-hexa­hydro-2*H*-indazol-2-yl]thia­zol-4(5*H*)-one

**DOI:** 10.1107/S2056989016002498

**Published:** 2016-02-17

**Authors:** Abdellah N’ait Ousidi, My Youssef Ait Itto, Aziz Auhmani, Abdelkhalek Riahi, Jean-Claude Daran, Auhmani Abdelwahed

**Affiliations:** a Laboratoire de Physico-Chimie Moléculaire et Synthèse Organique, Département de Chimie, Faculté des Sciences, Semlalia BP 2390, Marrakech 40001, Morocco; b Institut de Chimie Moléculaire de Reims, CNRS UMR 7312, Bat. Europol’Agr, Moulin de la Housse, UFR Sciences, BP 1039, 51687 Reims Cédex 2, France; c Laboratoire de Chimie de Coordination, CNRS UPR8241, 205 route de Narbonne, 31077 Toulouse Cedex 04, France

**Keywords:** crystal structure, absolute structure, heterocyclic compounds, thia­zolidinone, indazole, C—H⋯O hydrogen bonding, C—H⋯π inter­actions

## Abstract

The title compound, C_13_H_19_N_3_OS, is a new thia­zolidin-4-one derivative prepared and isolated as the pure (3a*S*,6*R*)-diastereisomer from (*R*)-thio­semicarbazone pulegone. It crystallized with two independent mol­ecules (*A* and *B*) in the asymmetric unit. The compound is composed of a hexhydro­indazole ring system (*viz.* a five-membered di­hydro­pyrazole ring fused to a cyclo­hexyl ring) with a thia­zole-4-one ring system attached to one of the pyrazole N atoms (at position 2). The overall geometry of the two mol­ecules differs slightly, with the mean planes of the pyrazole and thia­zole rings being inclined to one another by 10.4 (1)° in mol­ecule *A* and 0.9 (1)° in mol­ecule *B*. In the crystal, the *A* and *B* mol­ecules are linked *via* C—H⋯O hydrogen bonds, forming slabs parallel to the *ab* plane. There are C—H⋯π inter­actions present within the layers, and between the layers, so forming a three-dimensional structure.

## Abstract 2

The absolute structure of the title compound was determined from the synthetic pathway and by resonant scattering. The compound is a new thia­zolidin-4-one derivative, prepared from (*R*)-thio­semicarbazone pulegone, and was isolated on crystallization from ethanol as the pure (3a*S*,6*R*)-diastereisomer.

## Chemical context   

Thia­zolidinones constitute an important class of heterocyclic compounds containing sulfur and nitro­gen in a five-membered ring. They play a vital role due to their wide range of biological activities and industrial importance. Thia­zolidin-4-ones are particularly important because of their efficiency towards various pharmacological usages. A recent literature search reveals that thia­zolidin-4-one derivatives may exhibit anti­bacterial (Bonde & Gaikwad, 2004 ▸), anti­tuberculosis (Karali *et al.*, 2007 ▸), anti­viral (Kaushik-Basu *et al.*, 2008 ▸) and anti­cancer activities (Patel *et al.*, 2014 ▸).

As a part of our endeavour toward the preparation of new heterocyclic systems, we report herein on the structure of a new optically active thia­zolidin-4-one (**2**) synthesized from (*R*)-thio­semicarbazone pulegone (**1**); see Scheme. The reaction involves the treatment of thio­semicarbazone (**1**), in 
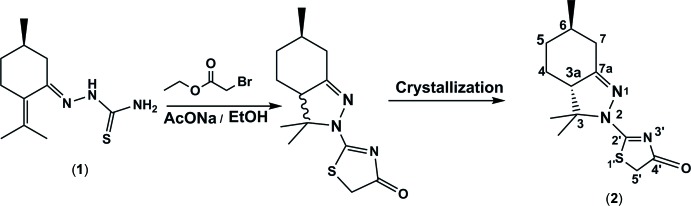
refluxing ethanol, with ethyl bromo­acatete and an excess of sodium acetate. Crystallization from an ethano­lic solution of the resulting indazolic thia­zolidin-4-one (obtained as a diastereomeric mixture) led to the isolation of compound (**2**). The structure of (**2**) was elucidated using spectroscopic (MS and NMR) data, while its absolute structure was determined as (3a*S*,6*R*) based mainly on the synthetic pathway and confirmed by resonant scattering.

## Structural commentary   

**Figure 1:**
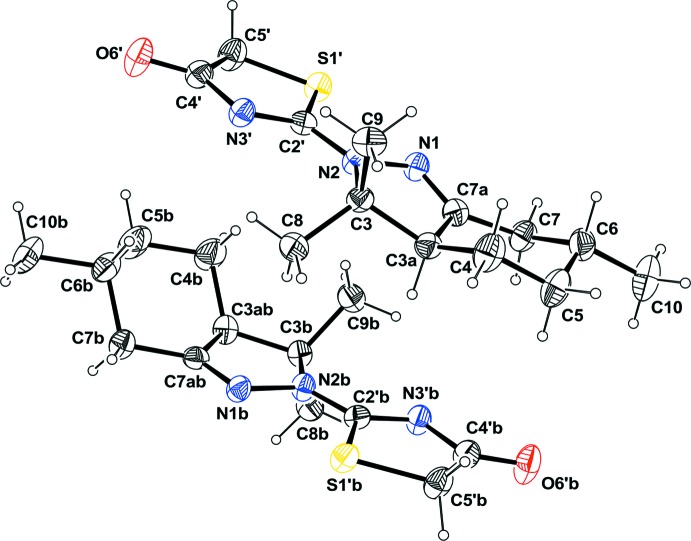
View of the mol­ecular structure of the two independent mol­ecules (*A* and *B*) of the title compound, showing the atom labelling. Displacement ellipsoids are drawn at the 50% probability level.

**Figure 2:**
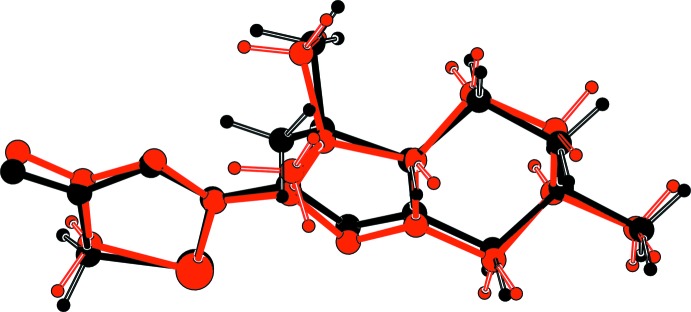
Mol­ecular fitting of independent mol­ecules *A* (black) and *B* (red).

The title compound crystallized with two independent mol­ecules (*A* and *B*) in the asymmetric unit. The compound is composed of a hexhydro­indazole ring system [*viz.* a five-membered di­hydro­pyrazole ring fused to a cyclo­hexyl ring] with a thia­zole-4-one ring system attached to pyrazole N atom N2 (Fig. 1 ▸). Mol­ecular fitting of the two mol­ecules (Spek, 2009 ▸) shows that they have roughly the same conformation and the same configuration (Fig. 2 ▸), even if some slight differences can be observed. The six-membered rings each display a chair conformation, with puckering parameters of θ = 12.96° and φ2 = 113.49° for mol­ecule *A* and θ = 9.44° and φ2 = 92.43° for mol­ecule *B*. The five-membered pyrazol rings are almost planar with the largest deviation being 0.081 (3) Å for atom C3 in mol­ecule *A* and −0.032 (1) for atom C3*B* in mol­ecule *B*. The thia­zole rings are planar, the largest deviation being −0.011 (1) Å for atom C2′ and 0.005 (1) for atom C5′*B* in mol­ecules *A* and *B*, respectively. In mol­ecule *A*, the two five-membered rings are slightly twisted with a dihedral angle of 10.4 (1)°, whereas in mol­ecule *B* the two rings are almost coplanar with a dihedral angle of 0.9 (1)°.

## Supra­molecular features   

**Figure 3:**
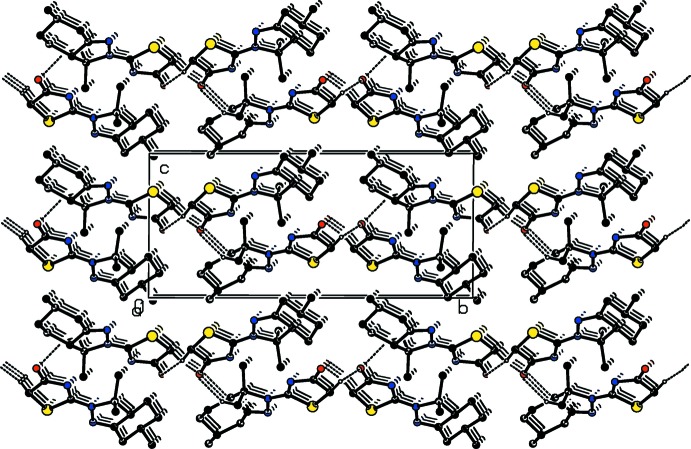
A view along the *a* axis of the crystal packing of the title compound, showing the formation of layers parallel to the *ab* plane *via* C—H⋯O hydrogen bonds (see Table 1 ▸). H atoms not involved in these inter­actions have been omitted for clarity.

**Figure 4:**
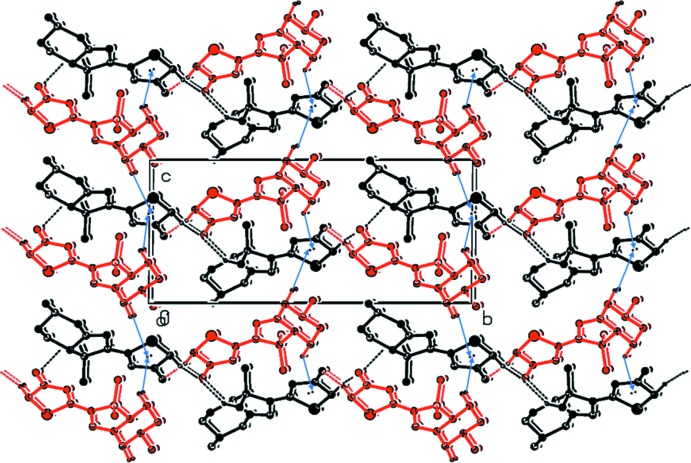
A view along the *a* axis of the crystal packing of the title compound, showing the C—H⋯O hydrogen bonds (dashed lines), and the C—H⋯π inter­actions (represented by blue arrows) linking the *A* (black) and *B* (red) mol­ecules within and between the layers (see Table 1 ▸). H atoms not involved in these inter­actions have been omitted for clarity.

In the crystal, the two independent mol­ecules are connected *via* C—H⋯O hydrogen bonds forming layers, or slabs, parallel to the *ab* plane (Table 1 ▸ and Fig. 3 ▸). Within the layers there are C—H⋯π inter­actions present (Fig. 4 ▸ and Table 1 ▸). The layers are also linked by C—H⋯π inter­actions (Table 1 ▸), forming a three-dimensional structure (Fig. 4 ▸).

## Database survey   

A search of the Cambridge Structural Database (CSD, V5.37, update November 2015; Groom & Allen, 2014 ▸) using the hexa­hydro­indazole ring system as the main skeleton, revealed the presence of 27 structures. A search for a thia­zole ring linked to an N atom of a pyrazole ring, similar to the situation in the title compound, yielded six hits. One of these structures, 2-(3-phenyl-3,3a,4,5-tetra­hydro-2*H*-benzo[*g*]indazol-2-yl)-1,3-thia­zol-4(5*H*)-one (refcode LUHGAY; Gautam & Chaudhary, 2015 ▸), resembles the title compound with an indazole ring system linked to a thia­zole ring. The mean plane of the two five-membered rings are inclined to one another by *ca* 10.05°, similar to the arrangement in mol­ecule *A* of the title compound.

## Synthesis and crystallization   

The synthesis of the title compound is illustrated in the Scheme. A mixture of thio­semicarbazone (**1**) (1.5 mmol, 1 eq), ethyl 2-bromo­acetate (0.24 ml, 1.5 mmol) and anhydrous sodium acetate (0.37 g, 4.5 mmol, 3 eq) in absolute ethanol (30 ml) was heated under reflux until the completion of the reaction (1–3 h). The solvent was then evaporated under reduced pressure and the crude product was purified by chromatography on silica gel (230–400 mesh) using hexa­ne/ethyl acetate (90:10) as eluent to give pure indazolic thia­zolidin-4-one in 60% yield as a diastereomeric mixture. Slow evaporation from an ethano­lic solution gives crystals of the pure diastereoisomer of the title compound (**2**) suitable for crystallographic analysis.

## Refinement   

Crystal data, data collection and structure refinement details are summarized in Table 2 ▸. The C-bound H atoms were included in calculated positions and treated as riding atoms: C—H = 0.96–0.98 Å with *U*_iso_(H) = 1.5*U*_eq_(C-meth­yl) and 1.2*U*_eq_(C) for other H atoms.

## Supplementary Material

Crystal structure: contains datablock(s) I, global. DOI: 10.1107/S2056989016002498/su5277sup1.cif

Structure factors: contains datablock(s) I. DOI: 10.1107/S2056989016002498/su5277Isup2.hkl

Supporting information file. DOI: 10.1107/S2056989016002498/su5277Isup3.cml

CCDC reference: 1452670

Additional supporting information:  crystallographic information; 3D view; checkCIF report

## Figures and Tables

**Table 1:** Hydrogen-bond geometry (Å, °) *Cg*1 is the centroid of the thia­zole ring S1′/N3′/C2′/C4′/C5′.

*D*—H⋯*A*	*D*—H	H⋯*A*	*D*⋯*A*	*D*—H⋯*A*
C5′—H5′2⋯O6′*B* ^i^	0.97	2.43	3.304 (4)	150
C9—H9*B*⋯O6′*B* ^ii^	0.96	2.53	3.361 (3)	145
C5′*B*—H5′3⋯O6′^iii^	0.97	2.44	3.361 (3)	159
C4*B*—H4*B*2⋯*Cg*1	0.96	2.93	3.737 (4)	141
C7*B*—H7*B*2⋯*Cg*1^iv^	0.96	2.90	3.867 (4)	174

Symmetry codes: (i) 
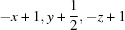
; (ii) 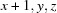; (iii) 
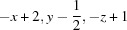
; (iv) 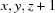.

**Table 2:** Experimental details

Crystal data	
Chemical formula	C_13_H_19_N_3_OS
*M*_r_	265.37
Crystal system, space group	Monoclinic, *P*2_1_
Temperature (K)	180
*a*, *b*, *c* (Å)	8.5519 (2), 18.9335 (4), 8.9165 (3)
β (°)	110.203 (3)
*V* (Å^3^)	1354.91 (7)
*Z*	4
Radiation type	Mo *K*α
μ (mm^−1^)	0.23
Crystal size (mm)	0.25 × 0.21 × 0.18

Data collection	
Diffractometer	Agilent Xcalibur Eos Gemini ultra
Absorption correction	Multi-scan (*CrysAlis PRO*; Agilent, 2014 ▸)
*T*_min_, *T*_max_	0.939, 1.000
No. of measured, independent and observed [*I* > 2σ(*I*)] reflections	15302, 6147, 5674
*R*_int_	0.024
(sin θ/λ)_max_ (Å^−1^)	0.692

Refinement	
*R*[*F* ^2^ > 2σ(*F* ^2^)], *wR*(*F* ^2^), *S*	0.033, 0.077, 1.04
No. of reflections	6147
No. of parameters	331
No. of restraints	1
H-atom treatment	H-atom parameters constrained
Δρ_max_, Δρ_min_ (e Å^−3^)	0.22, −0.19
Absolute structure	Flack *x* determined using 2349 quotients [(*I* ^+^)−(*I* ^-^)]/[(*I* ^+^)+(*I* ^−^)] (Parsons *et al.*, 2013 ▸)
Absolute structure parameter	−0.08 (3)

Computer programs: *CrysAlis PRO* (Agilent, 2014 ▸), *SIR97* (Altomare *et al.*, 1999 ▸), *SHELXL2013* (Sheldrick, 2015 ▸), *ORTEPIII* (Burnett & Johnson, 1996 ▸), *ORTEP-3 for Windows* (Farrugia, 2012 ▸) and *PLATON* (Spek, 2009 ▸).

## supplementary crystallographic information

### Crystal data

**Table:**

C_13_H_19_N_3_OS	*F*(000) = 568
*M**_r_* = 265.37	*D*_x_ = 1.301 Mg m^−^^3^
Monoclinic, *P*2_1_	Mo *K*α radiation, λ = 0.71073 Å
*a* = 8.5519 (2) Å	Cell parameters from 5859 reflections
*b* = 18.9335 (4) Å	θ = 3.6–29.2°
*c* = 8.9165 (3) Å	µ = 0.23 mm^−^^1^
β = 110.203 (3)°	*T* = 180 K
*V* = 1354.91 (7) Å^3^	Prismatic, colourless
*Z* = 4	0.25 × 0.21 × 0.18 mm

### Data collection

**Table:**

Agilent Xcalibur Eos Gemini ultra diffractometer	6147 independent reflections
Radiation source: Enhance (Mo) X-ray Source	5674 reflections with *I* > 2σ(*I*)
Graphite monochromator	*R*_int_ = 0.024
Detector resolution: 16.1978 pixels mm^-1^	θ_max_ = 29.5°, θ_min_ = 3.3°
ω scans	*h* = −11→11
Absorption correction: multi-scan (*CrysAlis PRO*; Agilent, 2014)	*k* = −23→25
*T*_min_ = 0.939, *T*_max_ = 1.000	*l* = −12→11
15302 measured reflections	

### Refinement

**Table:**

Refinement on *F*^2^	Hydrogen site location: inferred from neighbouring sites
Least-squares matrix: full	H-atom parameters constrained
*R*[*F*^2^ > 2σ(*F*^2^)] = 0.033	*w* = 1/[σ^2^(*F*_o_^2^) + (0.0364*P*)^2^ + 0.1555*P*] where *P* = (*F*_o_^2^ + 2*F*_c_^2^)/3
*wR*(*F*^2^) = 0.077	(Δ/σ)_max_ < 0.001
*S* = 1.04	Δρ_max_ = 0.22 e Å^−^^3^
6147 reflections	Δρ_min_ = −0.19 e Å^−^^3^
331 parameters	Absolute structure: Flack *x* determined using 2349 quotients [(*I*^+^)-(*I*^-^)]/[(*I*^+^)+(*I*^-^)] (Parsons *et al.*, 2013)
1 restraint	Absolute structure parameter: −0.08 (3)

### Special details

**Table:**

Geometry. All e.s.d.'s (except the e.s.d. in the dihedral angle between two l.s. planes) are estimated using the full covariance matrix. The cell e.s.d.'s are taken into account individually in the estimation of e.s.d.'s in distances, angles and torsion angles; correlations between e.s.d.'s in cell parameters are only used when they are defined by crystal symmetry. An approximate (isotropic) treatment of cell e.s.d.'s is used for estimating e.s.d.'s involving l.s. planes.

### Fractional atomic coordinates and isotropic or equivalent isotropic displacement parameters (Å^2^)

**Table:**

	*x*	*y*	*z*	*U*_iso_*/*U*_eq_	
S1'	0.77265 (7)	0.51418 (3)	0.26117 (7)	0.02894 (15)	
O6'	1.2287 (2)	0.54265 (10)	0.5307 (3)	0.0410 (5)	
N1	0.6528 (2)	0.37534 (10)	0.2176 (2)	0.0258 (4)	
N2	0.8216 (2)	0.37680 (10)	0.3163 (2)	0.0246 (4)	
N3'	1.0511 (3)	0.44868 (11)	0.4370 (2)	0.0269 (4)	
C2'	0.8954 (3)	0.43930 (12)	0.3469 (3)	0.0232 (5)	
C4'	1.0915 (3)	0.51929 (14)	0.4524 (3)	0.0290 (5)	
C5'	0.9500 (3)	0.56889 (15)	0.3626 (3)	0.0358 (6)	
H5'1	0.9808	0.5970	0.2862	0.043*	
H5'2	0.9245	0.6005	0.4366	0.043*	
C3	0.8934 (3)	0.30406 (12)	0.3685 (3)	0.0241 (5)	
C3A	0.7284 (3)	0.26108 (13)	0.3219 (3)	0.0266 (5)	
H3A	0.7035	0.2524	0.4197	0.032*	
C4	0.7166 (3)	0.19101 (16)	0.2363 (3)	0.0389 (6)	
H4A	0.7865	0.1564	0.3091	0.047*	
H4B	0.7564	0.1965	0.1474	0.047*	
C5	0.5357 (4)	0.16515 (15)	0.1746 (4)	0.0406 (7)	
H5A	0.5305	0.1200	0.1216	0.049*	
H5B	0.4984	0.1580	0.2645	0.049*	
C6	0.4191 (3)	0.21710 (15)	0.0582 (3)	0.0356 (6)	
H6	0.4574	0.2231	−0.0327	0.043*	
C7	0.4263 (3)	0.28925 (13)	0.1385 (3)	0.0318 (5)	
H7A	0.3692	0.3240	0.0583	0.038*	
H7B	0.3690	0.2864	0.2151	0.038*	
C7A	0.6020 (3)	0.31236 (12)	0.2218 (3)	0.0257 (5)	
C9	1.0058 (3)	0.28524 (14)	0.2749 (3)	0.0318 (5)	
H9A	0.9442	0.2890	0.1625	0.048*	
H9B	1.0454	0.2377	0.2997	0.048*	
H9C	1.0988	0.3171	0.3031	0.048*	
C8	0.9873 (3)	0.30082 (14)	0.5474 (3)	0.0331 (6)	
H8A	1.0870	0.3286	0.5732	0.050*	
H8B	1.0161	0.2527	0.5787	0.050*	
H8C	0.9182	0.3191	0.6033	0.050*	
C10	0.2398 (4)	0.1906 (2)	−0.0055 (4)	0.0551 (8)	
H10A	0.2358	0.1458	−0.0573	0.083*	
H10B	0.1715	0.2240	−0.0808	0.083*	
H10C	0.1990	0.1852	0.0816	0.083*	
S1'B	0.72290 (7)	0.19227 (3)	0.77175 (8)	0.02873 (15)	
O6'B	0.2793 (2)	0.16136 (10)	0.4764 (3)	0.0430 (5)	
N1B	0.8189 (2)	0.33287 (10)	0.8585 (2)	0.0256 (4)	
N2B	0.6558 (2)	0.32950 (10)	0.7482 (2)	0.0251 (4)	
N3'B	0.4417 (3)	0.25589 (11)	0.5972 (2)	0.0285 (4)	
C2'B	0.5933 (3)	0.26630 (13)	0.6977 (3)	0.0231 (5)	
C4'B	0.4099 (3)	0.18550 (14)	0.5654 (3)	0.0297 (5)	
C5'B	0.5556 (3)	0.13661 (14)	0.6521 (3)	0.0323 (6)	
H5'3	0.5899	0.1101	0.5756	0.039*	
H5'4	0.5236	0.1035	0.7194	0.039*	
C3B	0.5720 (3)	0.40092 (12)	0.7089 (3)	0.0243 (5)	
C3AB	0.7198 (3)	0.44937 (13)	0.8040 (3)	0.0272 (5)	
H3AB	0.6873	0.4763	0.8825	0.033*	
C4B	0.7936 (4)	0.50042 (17)	0.7135 (4)	0.0458 (7)	
H4B1	0.7139	0.5375	0.6651	0.055*	
H4B2	0.8175	0.4754	0.6290	0.055*	
C5B	0.9540 (4)	0.53296 (16)	0.8284 (4)	0.0483 (8)	
H5B1	0.9978	0.5662	0.7701	0.058*	
H5B2	0.9282	0.5590	0.9106	0.058*	
C6B	1.0865 (3)	0.47829 (14)	0.9071 (3)	0.0334 (6)	
H6B	1.1154	0.4540	0.8231	0.040*	
C7B	1.0200 (3)	0.42344 (13)	0.9955 (3)	0.0309 (5)	
H7B1	1.0964	0.3838	1.0262	0.037*	
H7B2	1.0117	0.4442	1.0919	0.037*	
C7AB	0.8528 (3)	0.39813 (13)	0.8913 (3)	0.0250 (5)	
C9B	0.5074 (3)	0.41293 (14)	0.5291 (3)	0.0328 (6)	
H9B1	0.4216	0.3791	0.4788	0.049*	
H9B2	0.5971	0.4075	0.4886	0.049*	
H9B3	0.4625	0.4598	0.5064	0.049*	
C8B	0.4338 (3)	0.40513 (15)	0.7789 (3)	0.0321 (6)	
H8B1	0.3807	0.4505	0.7553	0.048*	
H8B2	0.4799	0.3988	0.8926	0.048*	
H8B3	0.3533	0.3687	0.7330	0.048*	
C10B	1.2448 (3)	0.51173 (18)	1.0226 (4)	0.0468 (7)	
H10D	1.2862	0.5463	0.9669	0.070*	
H10E	1.3276	0.4758	1.0650	0.070*	
H10F	1.2202	0.5341	1.1085	0.070*	

### Atomic displacement parameters (Å^2^)

**Table:**

	*U*^11^	*U*^22^	*U*^33^	*U*^12^	*U*^13^	*U*^23^
S1'	0.0251 (3)	0.0232 (3)	0.0350 (3)	0.0039 (3)	0.0059 (3)	0.0028 (2)
O6'	0.0283 (10)	0.0288 (10)	0.0563 (12)	−0.0043 (8)	0.0025 (10)	−0.0012 (9)
N1	0.0204 (10)	0.0254 (10)	0.0294 (11)	0.0025 (8)	0.0058 (8)	−0.0001 (8)
N2	0.0189 (10)	0.0229 (10)	0.0299 (10)	0.0017 (8)	0.0059 (8)	0.0029 (8)
N3'	0.0226 (10)	0.0242 (10)	0.0311 (11)	0.0008 (8)	0.0057 (9)	0.0023 (8)
C2'	0.0254 (12)	0.0218 (12)	0.0237 (11)	0.0035 (9)	0.0103 (10)	0.0022 (9)
C4'	0.0276 (13)	0.0272 (13)	0.0320 (12)	−0.0002 (11)	0.0100 (11)	0.0012 (11)
C5'	0.0315 (14)	0.0259 (14)	0.0447 (15)	−0.0006 (12)	0.0065 (13)	0.0030 (11)
C3	0.0236 (11)	0.0207 (11)	0.0265 (12)	0.0019 (9)	0.0068 (10)	0.0049 (9)
C3A	0.0271 (12)	0.0266 (12)	0.0260 (11)	−0.0003 (10)	0.0089 (10)	0.0019 (10)
C4	0.0365 (15)	0.0238 (13)	0.0521 (17)	0.0023 (12)	0.0098 (13)	−0.0040 (13)
C5	0.0401 (16)	0.0269 (14)	0.0529 (17)	−0.0061 (12)	0.0138 (14)	−0.0103 (13)
C6	0.0362 (15)	0.0388 (14)	0.0319 (13)	−0.0079 (12)	0.0121 (12)	−0.0107 (12)
C7	0.0255 (12)	0.0298 (13)	0.0381 (14)	−0.0006 (10)	0.0083 (11)	−0.0009 (11)
C7A	0.0262 (12)	0.0255 (12)	0.0261 (12)	0.0021 (9)	0.0099 (10)	−0.0025 (9)
C9	0.0302 (13)	0.0313 (13)	0.0353 (13)	0.0074 (11)	0.0130 (11)	0.0061 (11)
C8	0.0343 (14)	0.0337 (14)	0.0273 (13)	−0.0018 (11)	0.0055 (11)	0.0051 (10)
C10	0.0411 (17)	0.0507 (19)	0.064 (2)	−0.0136 (17)	0.0065 (16)	−0.0210 (17)
S1'B	0.0231 (3)	0.0223 (3)	0.0364 (3)	0.0027 (2)	0.0046 (3)	0.0057 (2)
O6'B	0.0303 (11)	0.0298 (10)	0.0540 (12)	−0.0032 (9)	−0.0044 (10)	−0.0025 (9)
N1B	0.0193 (9)	0.0254 (11)	0.0285 (10)	−0.0003 (8)	0.0037 (8)	0.0039 (8)
N2B	0.0201 (10)	0.0224 (10)	0.0283 (10)	0.0031 (8)	0.0025 (8)	0.0012 (8)
N3'B	0.0249 (11)	0.0225 (10)	0.0324 (11)	0.0010 (9)	0.0025 (9)	0.0008 (9)
C2'B	0.0215 (12)	0.0232 (12)	0.0254 (11)	0.0024 (9)	0.0089 (10)	0.0034 (9)
C4'B	0.0268 (13)	0.0262 (13)	0.0335 (13)	−0.0005 (11)	0.0072 (11)	0.0009 (11)
C5'B	0.0287 (14)	0.0204 (13)	0.0422 (15)	0.0004 (11)	0.0054 (12)	0.0033 (11)
C3B	0.0243 (12)	0.0206 (11)	0.0256 (12)	0.0048 (9)	0.0056 (10)	0.0000 (9)
C3AB	0.0257 (12)	0.0250 (12)	0.0306 (13)	0.0032 (9)	0.0093 (10)	−0.0053 (10)
C4B	0.0447 (17)	0.0318 (16)	0.0514 (18)	−0.0054 (12)	0.0043 (14)	0.0139 (13)
C5B	0.0503 (18)	0.0270 (15)	0.062 (2)	−0.0110 (13)	0.0127 (16)	0.0076 (13)
C6B	0.0315 (14)	0.0320 (14)	0.0414 (15)	−0.0078 (11)	0.0187 (12)	−0.0081 (11)
C7B	0.0247 (12)	0.0320 (13)	0.0341 (13)	−0.0027 (10)	0.0077 (10)	−0.0014 (11)
C7AB	0.0260 (12)	0.0281 (12)	0.0230 (11)	−0.0009 (10)	0.0111 (10)	0.0021 (9)
C9B	0.0398 (14)	0.0280 (13)	0.0285 (13)	0.0026 (11)	0.0090 (12)	0.0000 (10)
C8B	0.0262 (12)	0.0385 (14)	0.0315 (13)	0.0035 (11)	0.0100 (11)	−0.0004 (11)
C10B	0.0370 (16)	0.0417 (17)	0.0629 (19)	−0.0179 (15)	0.0190 (15)	−0.0129 (16)

### Geometric parameters (Å, º)

**Table:**

S1'—C2'	1.772 (2)	S1'B—C2'B	1.767 (2)
S1'—C5'	1.801 (3)	S1'B—C5'B	1.800 (3)
O6'—C4'	1.222 (3)	O6'B—C4'B	1.214 (3)
N1—C7A	1.274 (3)	N1B—C7AB	1.280 (3)
N1—N2	1.408 (3)	N1B—N2B	1.404 (3)
N2—C2'	1.324 (3)	N2B—C2'B	1.324 (3)
N2—C3	1.514 (3)	N2B—C3B	1.513 (3)
N3'—C2'	1.308 (3)	N3'B—C2'B	1.312 (3)
N3'—C4'	1.376 (3)	N3'B—C4'B	1.370 (3)
C4'—C5'	1.523 (4)	C4'B—C5'B	1.531 (3)
C5'—H5'1	0.9700	C5'B—H5'3	0.9700
C5'—H5'2	0.9700	C5'B—H5'4	0.9700
C3—C9	1.517 (3)	C3B—C8B	1.517 (3)
C3—C8	1.519 (3)	C3B—C9B	1.522 (3)
C3—C3A	1.556 (3)	C3B—C3AB	1.555 (3)
C3A—C7A	1.497 (3)	C3AB—C7AB	1.493 (3)
C3A—C4	1.517 (4)	C3AB—C4B	1.529 (4)
C3A—H3A	0.9800	C3AB—H3AB	0.9800
C4—C5	1.532 (4)	C4B—C5B	1.529 (4)
C4—H4A	0.9700	C4B—H4B1	0.9700
C4—H4B	0.9700	C4B—H4B2	0.9700
C5—C6	1.524 (4)	C5B—C6B	1.516 (4)
C5—H5A	0.9700	C5B—H5B1	0.9700
C5—H5B	0.9700	C5B—H5B2	0.9700
C6—C10	1.525 (4)	C6B—C10B	1.527 (4)
C6—C7	1.534 (4)	C6B—C7B	1.528 (3)
C6—H6	0.9800	C6B—H6B	0.9800
C7—C7A	1.493 (3)	C7B—C7AB	1.490 (3)
C7—H7A	0.9700	C7B—H7B1	0.9700
C7—H7B	0.9700	C7B—H7B2	0.9700
C9—H9A	0.9600	C9B—H9B1	0.9600
C9—H9B	0.9600	C9B—H9B2	0.9600
C9—H9C	0.9600	C9B—H9B3	0.9600
C8—H8A	0.9600	C8B—H8B1	0.9600
C8—H8B	0.9600	C8B—H8B2	0.9600
C8—H8C	0.9600	C8B—H8B3	0.9600
C10—H10A	0.9600	C10B—H10D	0.9600
C10—H10B	0.9600	C10B—H10E	0.9600
C10—H10C	0.9600	C10B—H10F	0.9600
			
C2'—S1'—C5'	88.45 (12)	C2'B—S1'B—C5'B	88.60 (12)
C7A—N1—N2	106.63 (19)	C7AB—N1B—N2B	107.21 (19)
C2'—N2—N1	117.31 (18)	C2'B—N2B—N1B	117.75 (19)
C2'—N2—C3	129.48 (19)	C2'B—N2B—C3B	128.74 (19)
N1—N2—C3	113.19 (17)	N1B—N2B—C3B	113.44 (18)
C2'—N3'—C4'	111.1 (2)	C2'B—N3'B—C4'B	111.5 (2)
N3'—C2'—N2	124.0 (2)	N3'B—C2'B—N2B	123.7 (2)
N3'—C2'—S1'	118.80 (18)	N3'B—C2'B—S1'B	118.70 (18)
N2—C2'—S1'	117.20 (17)	N2B—C2'B—S1'B	117.56 (17)
O6'—C4'—N3'	124.6 (2)	O6'B—C4'B—N3'B	125.0 (2)
O6'—C4'—C5'	120.6 (2)	O6'B—C4'B—C5'B	120.5 (2)
N3'—C4'—C5'	114.8 (2)	N3'B—C4'B—C5'B	114.5 (2)
C4'—C5'—S1'	106.73 (19)	C4'B—C5'B—S1'B	106.68 (18)
C4'—C5'—H5'1	110.4	C4'B—C5'B—H5'3	110.4
S1'—C5'—H5'1	110.4	S1'B—C5'B—H5'3	110.4
C4'—C5'—H5'2	110.4	C4'B—C5'B—H5'4	110.4
S1'—C5'—H5'2	110.4	S1'B—C5'B—H5'4	110.4
H5'1—C5'—H5'2	108.6	H5'3—C5'B—H5'4	108.6
N2—C3—C9	108.11 (18)	N2B—C3B—C8B	109.00 (19)
N2—C3—C8	111.76 (19)	N2B—C3B—C9B	110.36 (19)
C9—C3—C8	111.3 (2)	C8B—C3B—C9B	112.0 (2)
N2—C3—C3A	99.17 (17)	N2B—C3B—C3AB	99.81 (18)
C9—C3—C3A	114.7 (2)	C8B—C3B—C3AB	110.28 (19)
C8—C3—C3A	111.2 (2)	C9B—C3B—C3AB	114.7 (2)
C7A—C3A—C4	111.0 (2)	C7AB—C3AB—C4B	107.9 (2)
C7A—C3A—C3	102.81 (19)	C7AB—C3AB—C3B	103.32 (19)
C4—C3A—C3	119.2 (2)	C4B—C3AB—C3B	119.4 (2)
C7A—C3A—H3A	107.8	C7AB—C3AB—H3AB	108.6
C4—C3A—H3A	107.8	C4B—C3AB—H3AB	108.6
C3—C3A—H3A	107.8	C3B—C3AB—H3AB	108.6
C3A—C4—C5	110.1 (2)	C3AB—C4B—C5B	109.9 (2)
C3A—C4—H4A	109.6	C3AB—C4B—H4B1	109.7
C5—C4—H4A	109.6	C5B—C4B—H4B1	109.7
C3A—C4—H4B	109.6	C3AB—C4B—H4B2	109.7
C5—C4—H4B	109.6	C5B—C4B—H4B2	109.7
H4A—C4—H4B	108.2	H4B1—C4B—H4B2	108.2
C6—C5—C4	112.3 (2)	C6B—C5B—C4B	112.9 (3)
C6—C5—H5A	109.1	C6B—C5B—H5B1	109.0
C4—C5—H5A	109.1	C4B—C5B—H5B1	109.0
C6—C5—H5B	109.1	C6B—C5B—H5B2	109.0
C4—C5—H5B	109.1	C4B—C5B—H5B2	109.0
H5A—C5—H5B	107.9	H5B1—C5B—H5B2	107.8
C5—C6—C10	112.2 (3)	C5B—C6B—C10B	112.1 (2)
C5—C6—C7	110.2 (2)	C5B—C6B—C7B	110.5 (2)
C10—C6—C7	109.9 (2)	C10B—C6B—C7B	109.6 (2)
C5—C6—H6	108.1	C5B—C6B—H6B	108.2
C10—C6—H6	108.1	C10B—C6B—H6B	108.2
C7—C6—H6	108.1	C7B—C6B—H6B	108.2
C7A—C7—C6	111.4 (2)	C7AB—C7B—C6B	110.2 (2)
C7A—C7—H7A	109.4	C7AB—C7B—H7B1	109.6
C6—C7—H7A	109.4	C6B—C7B—H7B1	109.6
C7A—C7—H7B	109.4	C7AB—C7B—H7B2	109.6
C6—C7—H7B	109.4	C6B—C7B—H7B2	109.6
H7A—C7—H7B	108.0	H7B1—C7B—H7B2	108.1
N1—C7A—C7	123.6 (2)	N1B—C7AB—C7B	123.1 (2)
N1—C7A—C3A	116.2 (2)	N1B—C7AB—C3AB	115.9 (2)
C7—C7A—C3A	120.1 (2)	C7B—C7AB—C3AB	120.7 (2)
C3—C9—H9A	109.5	C3B—C9B—H9B1	109.5
C3—C9—H9B	109.5	C3B—C9B—H9B2	109.5
H9A—C9—H9B	109.5	H9B1—C9B—H9B2	109.5
C3—C9—H9C	109.5	C3B—C9B—H9B3	109.5
H9A—C9—H9C	109.5	H9B1—C9B—H9B3	109.5
H9B—C9—H9C	109.5	H9B2—C9B—H9B3	109.5
C3—C8—H8A	109.5	C3B—C8B—H8B1	109.5
C3—C8—H8B	109.5	C3B—C8B—H8B2	109.5
H8A—C8—H8B	109.5	H8B1—C8B—H8B2	109.5
C3—C8—H8C	109.5	C3B—C8B—H8B3	109.5
H8A—C8—H8C	109.5	H8B1—C8B—H8B3	109.5
H8B—C8—H8C	109.5	H8B2—C8B—H8B3	109.5
C6—C10—H10A	109.5	C6B—C10B—H10D	109.5
C6—C10—H10B	109.5	C6B—C10B—H10E	109.5
H10A—C10—H10B	109.5	H10D—C10B—H10E	109.5
C6—C10—H10C	109.5	C6B—C10B—H10F	109.5
H10A—C10—H10C	109.5	H10D—C10B—H10F	109.5
H10B—C10—H10C	109.5	H10E—C10B—H10F	109.5

### Hydrogen-bond geometry (Å, º)

Cg1 is the centroid of the thiazole ring S1'/N3'/C2'/C4'/C5'

**Table:**

*D*—H···*A*	*D*—H	H···*A*	*D*···*A*	*D*—H···*A*
C5′—H5′2···O6′*B*^i^	0.97	2.43	3.304 (4)	150
C9—H9*B*···O6′*B*^ii^	0.96	2.53	3.361 (3)	145
C5′*B*—H5′3···O6′^iii^	0.97	2.44	3.361 (3)	159
C4*B*—H4*B*2···*Cg*1	0.96	2.93	3.737 (4)	141
C7*B*—H7*B*2···*Cg*1^iv^	0.96	2.90	3.867 (4)	174

Symmetry codes: (i) −*x*+1, *y*+1/2, −*z*+1; (ii) *x*+1, *y*, *z*; (iii) −*x*+2, *y*−1/2, −*z*+1; (iv) *x*, *y*, *z*+1.
